# Motor dysfunction and touch-slang in user interface data

**DOI:** 10.1038/s41598-017-04893-1

**Published:** 2017-07-05

**Authors:** Yoni Klein, Ruth Djaldetti, Yosi Keller, Ido Bachelet

**Affiliations:** 1 Faculty of Engineering, Bar-Ilan University, Ramat-Gan, Israel; 2 Department of Neurology, Rabin Medical Center, Beilinson Hospital, Petach Tikva; and Sackler Faculty of Medicine, Tel Aviv University, Tel Aviv, Israel; 3 Augmanity, Rehovot, Israel

## Abstract

The recent proliferation in mobile touch-based devices paves the way for increasingly efficient, easy to use natural user interfaces (NUI). Unfortunately, touch-based NUIs might prove difficult, or even impossible to operate, in certain conditions e.g. when suffering from motor dysfunction such as Parkinson’s Disease (PD). Yet, the prevalence of such devices makes them particularly suitable for acquiring motor function data, and enabling the early detection of PD symptoms and other conditions. In this work we acquired a unique database of more than 12,500 annotated NUI multi-touch gestures, collected from PD patients and healthy volunteers, that were analyzed by applying advanced shape analysis and statistical inference schemes. The proposed analysis leads to a novel detection scheme for early stages of PD. Moreover, our computational analysis revealed that young subjects may be using a ‘slang’ form of gesture-making to reduce effort and attention cost while maintaining meaning, whereas older subjects put an emphasis on content and precise performance.

## Introduction

The search for increasingly efficient, easy to master human-machine interfaces (HMI), have led to the minimization of the interface space and adherence to innate and natural motions, where early HMI utilized the manipulation of graphical objects by light pens (in Ivan Sutherland’s Sketchpad)^1^ or a clickable mouse^2^.

In contrast, contemporary HMIs utilize *touch-based* natural user interfaces (NUI), that have become ubiquitous in smartphones and tablet computers. However, such NUIs might induce significant difficulties when operated by users whose fine motor abilities are compromised, as their operation requires specific gestures within pre-defined precision margins, particularly when applying multi-finger gestures.

NUI gesturing is particularly compromised due to motor dysfunction disorders - a class of medical conditions in which afflicted individuals are unable to plan or execute motions precisely, or even at all. The causative factors are diverse and include injuries, diseases of the motor cortex, motor neurons, skeletal muscles, and neurodegenerative disorders.

The rate of medical conditions such as Parkinson disease (PD) or multiple sclerosis is increasing in developed countries, due to the increase in life expectancy, and as touch phones and their apps are an essential part of modern life, relating to messaging (Whatsapp), social networks (Facebook) and services (Uber etc.), this issue becomes a significant problem for the elderly community.

PD is a neurodegenerative disorder affecting the basal ganglia in the central nervous system^3^. The prominent motor symptoms associated with PD are a result of gradual death of dopaminergic neurons in the substantia nigra (SN), a process that is still unclear. The motor symptoms that are often the first sign leading to the diagnosis of PD, occur at a relatively late stage of the process, where as high as 75% of SN cells are lost^4^. It is estimated to affect more than 10 million people worldwide^5^, making its early detection crucial.

Thus, daily-performed fine motions, such as handwriting or keyboard writing, generate a constant stream of data that could be used to monitor the quality of motor functions. For instance, a gradual increase of typing errors followed by ‘DELETE’ key strokes, observed over a long period of time, could suggest declining motor functionality. It has also been suggested to use EMG signals produced while handwriting, to distinguish between subjects with PD and healthy subjects^6^.

The increasing proliferation of touch-based NUI personal devices, such as cellphone, makes them an ideal mean for collecting motor function data, analyzing and monitoring it. Thus, enabling early detection of abnormal signs. In this study we aim to study this hypothesis using standard gestures required to operate touch NUI, and to acquire a novel proprietary dataset for studying the detection of motor dysfunction, at an early disease stage as possible.

To this end we acquired a unique novel database of more than 12,500 annotated NUI multi-touch gestures collected from PD patients and healthy volunteers, and designed a pattern recognition algorithm based on machine learning and computational shape analysis to retrieve the patterns directly from the acquired gestures. By constantly monitoring the quality of executed gestures and comparing it to past gestures, the proposed computational scheme could identify statistical patterns that indicate an ongoing decline of motor functions and prompt the need for immediate physical examination at an early disease stage.

Interestingly, we discovered an unexpected similarity between the gestures made by PD and young subjects, where subsequent statistical analysis showed that young subjects tend to a form of ‘touch-slang’, cutting down effort and attention in gesturing to maintain meaning, while PD patients demonstrate a similar phenotype due to their disability, and these implications are shown and discussed.

## Methods

### Data collection

We collected multi-touch gestures from healthy young volunteers (n = 33, mean age 26.8 ± 4.6 years), healthy elderly volunteers (n = 14, mean age 79.9 ± 7.7 years), and PD patients (n = 27, mean age 66.9 ± 7.9 years). At the time of collection, all subjects have been using a touch interface-based device for at least 3 years and testified to be proficient in its operation. PD diagnosis was based on establishing 2 out of 3 disease criteria (rest tremor, bradykinesia, and muscular rigidity). PD group subjects were diagnosed with PD not more than 3 to 5 years prior to data collection. Patients with longer periods since diagnosis were excluded from this study. Healthy elderly subjects were all in excellent cognitive, motor, and functional state.

Each subject was asked to perform 18 different gestures, 10 repeats or more per gesture. The following gestures were used: one finger scroll, four finger scale, two finger scroll, three finger scale, four finger scroll, one finger drag, two finger horizontal scale, two finger drag, Two finger vertical scale, Three finger scroll, Two finger rotate (single hand), Three finger rotate, Lock 2 and one finger vertical tilt, two finger rotate (two hands), Four finger drag, Three finger drag, Two finger split, Lock 2 and one finger horizontal tilt. The gestures were collected using an Android Samsung Galaxy 10.1 tablet, and a proprietary, specially implemented acquisition application (Available at: https://drive.google.com/open?id=0B20J1SUHMkFWX0w0UUd1ak1XNDQ).

The experimental protocol and the informed consent form and explanation were reviewed and approved by the Institutional Helsinki Review Board of Rabin Medical Center for experiments involving human subjects, and all methods were performed in accordance with the relevant guidelines and regulations. The purpose and procedure of the experiment were communicated to the subjects, who signed consent forms prior to participating.

### Algorithm and data analysis

Each gesture was encoded using the inner-distance shape context (IDSC)^7^, that is a descriptor that encodes a shape by storing the distances of each point on the shape to the other points on the shape, with respect to the relative angles between each pair of points. The IDSC descriptor was shown to be robust to shape articulations in general and rotations in particular.

Spectral graph matching^8^ was applied to quantify the similarity between pairs of shapes, being robust to the varying number of points in each shape and relative rotations and translations. The scale of the shapes is normalized by computing the Principle Component Analysis (PCA) of the set of points encoding each shape and normalizing by the PCA’s largest eigenvalue.

The shape similarity scores of all 18 shapes of a particular subject were stacked in a single vector, to utilize all classification cues including subject-derived patterns that are common across all shapes. This representation allows to train a support vector machine (SVM) model to classify the test set data as healthy/non-healthy, where ten fold Cross Validation (CV) was applied to avoid overfitting. The detection performance was quantified using Receiver Operation Characteristic (ROC) curves, and the areas under curve (AUC) were calculated per shape, where the results of the 10 CV trials were averaged to evaluate the model performance.

## Results

**Figure 1:**
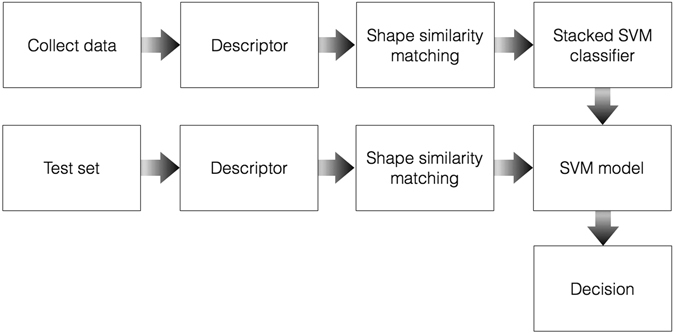
Block diagram of the algorithm.

In this work we derived a computational approach for analyzing touch gestures, that is applicable to detecting statistical patterns of motor dysfunction, by utilizing computational shape similarity. For that we applied supervised stacking-based SVM classification (Fig. 1), to 12,500 fully annotated gestures acquired using a proprietary android application we developed, while storing all data points per shape and their temporal time stamps, used to estimate the writing velocity and acceleration.

We used the 18, previously detailed, gestures as these are part of an existing, easily acquired shape vocabulary and are commonly used in the context of touch-based devices. It follows that the most discriminative shapes were those that require the operation of four fingers or more, shapes requiring movements in various directions simultaneously, or inconsistent operation of each hand. Shapes based on single finger movements such as one finger scroll or drag, showed similar performance score among all groups. Interestingly, gestures requiring two fingers being held tightly together, were shown to be non-discriminative as they relate to coarser, more easily-controlled motions.

**Figure 2:**
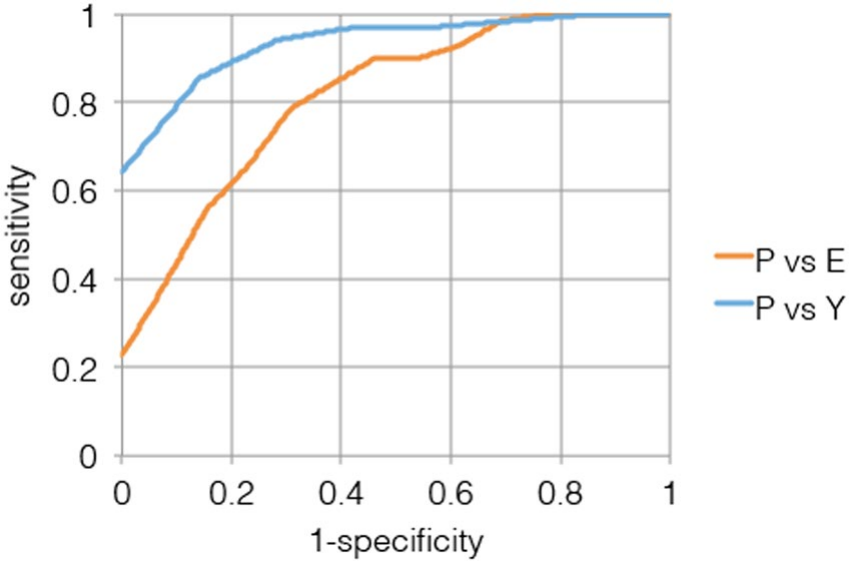
Algorithm discrimination performance. ROC curves of Young vs. PD and Elderly vs. PD.

The proposed scheme is able to effectively detect PD patients as depicted in the ROC curves in Fig. 2, where it follows that high sensitivity rates of ~90% can be achieved. This exemplifies the validity of the core algorithmic result of our work, that is the computational detection of PD, while paving the way for applying the proposed scheme s as a preemptive real-time PD detection measure.

We also utilized the proposed healthy/non-healthy classification scheme (Fig. 2), as a mean to estimate the conceptual distances between the different classes of subjects. For that we compared the classification margins computed when aiming to discriminate between different classes. It follows that the classification margin between elderly subjects and PD patients is larger than that between young subjects and PD patients, implying, rather Counter-intuitively, that PD patients are more similar to young subjects than to older ones, in terms of their touch screen gestures.

**Figure 3:**
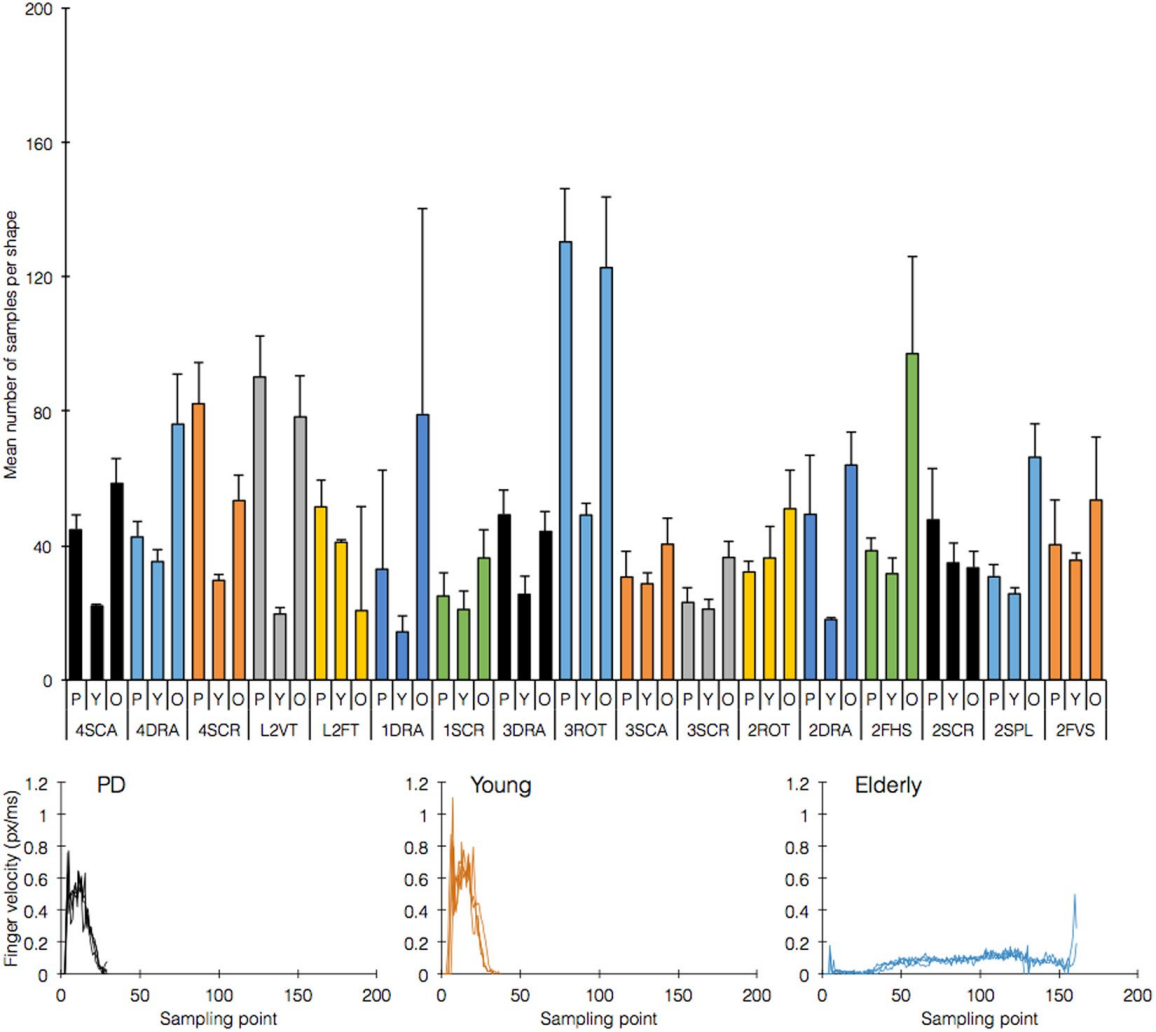
Similarity distribution between PD, young, and elderly subjects according to shape. The figure reports the mean count of data points per shape. Shape acronyms: 1SCR = one finger scroll, 4SCA = four finger scale, 2SCR = two finger scroll, 3SCA = three finger scale, 4SCR = four finger scroll, 1DRA = one finger drag, 2FHS = two finger horizontal scale, 2DRA = two finger drag, 2FVS = two finger vertical scale, 3SCR = three finger scroll, 3ROT = three finger rotate, L2VT = lock 2 and one finger vertical tilt, 2ROT = two finger rotate [two hand], 4DRA = four finger drag, 3DRA = three finger drag, 2SPL = two finger split, L2FT = lock 2 and one finger horizontal tilt (shown here are 17/18 shapes; two finger rotate [one hand] is on a different scale). Bottom panels show overlaid finger traces from a representative shape, showing PD-Y similarity.

We attribute that to young subjects striving to minimize the cost of attention and effort required to make precise gestures, while maintaining their meaning. In contrast, older subjects aimed to produce more precise gestures, at the cost of higher efforts. This is an interesting equivalent to *slang* in spoken languages, which is a language dialect that utilizes very flexible syntax and content, overlooking proper formal grammar. Specifically, PD patients were more similar to elderly patients in 6/18 of the shapes, while being more similarity to young subjects in 9/18 of the shapes (Fig. 3). In particular, as young subjects use the gestures slang by choice, some of them in our dataset used exact gestures, implying that the similarity we observe due to the slang phenomenon is more statistically significant.

**Figure 4:**
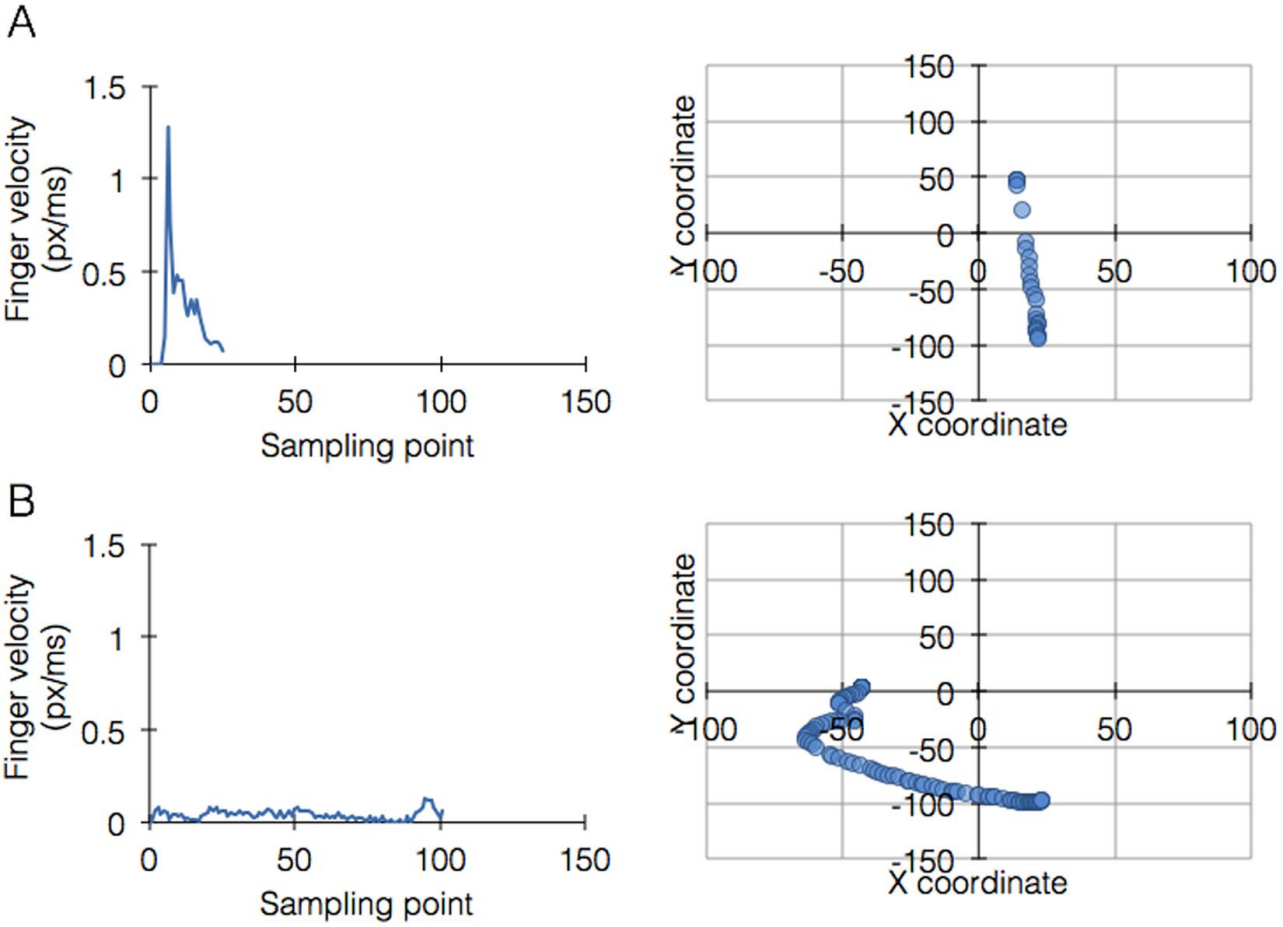
Metadata analyses showing differential finger performance. Representative single finger trace data from a complex gesture (in this case 4 finger scale). (**A**) Finger performing as normally expected: fewer high velocity data points at impact with screen, relieving towards the end with many slower data points at the end. (**B**) stiff finger showing a significantly longer gesturing time, uniform low velocity throughout and uniform distribution of data points.

We also observed patterns of interest, as finger stiffness and coordination can be extracted by analyzing the velocity of each finger during a gesture. At the start of a gesture (t = 0), the fingers produce a smooth motion, with less data points, that are wider apart due to the interaction between the finger and the screen. The motion starts with high velocity but slows down towards the end, with more data points near the end (t = end). In contrast, stiff fingers show uniform distribution of touch points during a much longer gesturing time as depicted in Fig. 4.

## Discussion

The underlying motivation of our study is the double-edged sword of touch screens in contemporary digital society. On the on hand, these devices are ubiquitous and can be used to automatically and efficiently gather valuable information, while on the other hand, as traditional services and applications such as taxis (Uber, Lift stc.), and interpersonal communications (Whatsapp, Facebook etc.) migrate to mobile devices, might cause significant disadvantage to those unable to operate them.

In particular, due to the increased life expectancy, resulting in an ever increasing prevalence of neurodegenerative diseases such as PD, Alzheimer’s disease, and related conditions, one can expect these issues to become widespread, as current generations that are used to, and dependent on touch-based devices, become older.

For that we acquired a novel dataset, that to the best of our knowledge, is the first large annotated database of touch-based gestures that includes healthy young, healthy elderly, and PD subjects. Previously related databases, such as the Kaggle database^9, 10^, are either poorly annotated, of smaller scale, or contain custom-designed shapes, and therefore cannot be used to derive a classifier capable of using everyday gestures for diagnostic purposes.

Using the gestures dataset we derived a computational approach for analyzing touch-based gestures, that allows to compute the similarity between pairs of gestures, and thus use the similarity scores for learning to classify PD patients. In that, we addressed two challenges: first, being able to classify (identify), with high probability, a subject as a possible PD patient, based on his touch-based gestures, and second, we statistically analyzed the classification results to show that for some gestures young subjects tend to use a particular “slang” of gestures, that is a simplified and less manually demanding replica of the gestures.

Care was taken in this study to avoid biases not associated with the dysfunctional motor patterns of PD. In particular, we wished to eliminate variations in the ‘digital literacy’ of the subjects – their proficiency in operating touch interfaces – which is arguably higher in younger subjects. In this regard, it is important to distinguish between ‘digital natives’, ‘digital immigrants’, and ‘digital illiterates’, reflecting the different levels of operative proficiency. Since at present, wide use of touch interfaces as a phenomenon has begun approximately 10 years ago, it can be claimed that all individuals over a certain age threshold are digital immigrants, including all subjects who participated in this study (the youngest of which were ~22 years old). To account for this potential bias, subjects who were not proficient operators of touch interfaces with over 3 years of experience, were excluded from this study. Surprisingly, we encountered young individuals who intentionally maintained digital illiteracy for personal reasons, leading to their exclusion from participation.

One potential drawback in the experimental design is the mean age difference between the PD and healthy elderly groups. This difference resulted from excluding older patients for being diagnosed with PD more than 5 years prior to data collection, likely reflecting later stages of PD which we wished to exclude from our dataset. Although based on mean age, the PD group (~67 years) is still clustered together with the elderly group (~80 years) compared with the young group (~27 years), it is a potential source of bias and has to be further studied. We have designed a wider collection scheme aiming to complement the current dataset and minimize this difference to a minimum.

In future, our algorithm could be applied by touch-based devices to correct coarse “slang” gestures to produce the desired outcome, and monitor gesture performance over time to flag signs of potential decline in motor function. Moreover, the metadata collected by the algorithm layer could indicate important long-term trends in other physical and cognitive functions. For instance, consistent increase of scale of the gestures could indicate vision impairment.
